# Diagnostic Accuracy and Stability of Multimodal Large Language Models for Hand Fracture Detection: A Multi-Run Evaluation on Plain Radiographs

**DOI:** 10.3390/diagnostics16030424

**Published:** 2026-02-01

**Authors:** Ibrahim Güler, Gerrit Grieb, Armin Kraus, Martin Lautenbach, Henrik Stelling

**Affiliations:** 1 Department of Plastic, Aesthetic and Hand Surgery, Otto-von-Guericke University, 39120 Magdeburg, Germany; armin.kraus@med.ovgu.de; 2 Department of Health Management, Friedrich-Alexander-Universität Erlangen-Nürnberg (FAU), Lange Gasse 20, 90403 Nürnberg, Germany; 3 Department of Plastic Surgery and Hand Surgery, Gemeinschaftskrankenhaus Havelhoehe, Kladower Damm 221, 14089 Berlin, Germany; gerritgrieb@gmx.de; 4 Department of Plastic Surgery and Hand Surgery, Burn Center, Medical Faculty, RWTH Aachen University, Pauwelsstrasse 30, 52074 Aachen, Germany; 5 Department of Hand Surgery, Orthopedics, and Trauma Surgery, Waldfriede Hospital, Argentinische Allee 40, 14163 Berlin, Germany; m.lautenbach@waldfriede.de; 6 Practices for Nuclear Medicine, Rubensstraße 125, 12157 Berlin, Germany; henrikstelling@googlemail.com

**Keywords:** multimodal large language models, hand fractures, radiography, diagnostic accuracy, inter-run reliability, artificial intelligence, fracture detection, medical imaging, exploratory analysis, hand surgery

## Abstract

**Background/Objectives**: Multimodal large language models (MLLMs) offer potential for automated fracture detection, yet their diagnostic stability under repeated inference remains underexplored. This study evaluates the diagnostic accuracy, stability, and intra-model consistency of four MLLMs in detecting hand fractures on plain radiographs. **Methods**: In total, images of hand radiographs of 65 adult patients with confirmed hand fractures (30 phalangeal, 30 metacarpal, 5 scaphoid) were evaluated by four models: GPT-5 Pro, Gemini 2.5 Pro, Claude Sonnet 4.5, and Mistral Medium 3.1. Each image was independently analyzed five times per model using identical zero-shot prompts (1300 total inferences). Diagnostic accuracy, inter-run reliability (Fleiss’ κ), case-level agreement profiles, subgroup performance, and exploratory demographic inference (age, sex) were assessed. **Results**: GPT-5 Pro achieved the highest accuracy (64.3%) and consistency (κ = 0.71), followed by Gemini 2.5 Pro (56.9%, κ = 0.57). Mistral Medium 3.1 exhibited high agreement (κ = 0.88) despite low accuracy (38.5%), indicating systematic error (“confident hallucination”). Claude Sonnet 4.5 showed low accuracy (33.8%) and consistency (κ = 0.33), reflecting instability. While phalangeal fractures were reliably detected by top models, scaphoid fractures remained challenging. Demographic analysis revealed poor capabilities, with age estimation errors exceeding 12 years and sex prediction accuracy near random chance. **Conclusions**: Diagnostic accuracy and consistency are distinct performance dimensions; high intra-model agreement does not imply correctness. While GPT-5 Pro demonstrated the most favorable balance of accuracy and stability, other models exhibited critical failure modes ranging from systematic bias to random instability. At present, MLLMs should be regarded as experimental diagnostic reasoning systems rather than reliable standalone tools for clinical fracture detection.

## 1. Introduction

Hand fractures represent one of the most frequent musculoskeletal injuries encountered in emergency departments and orthopedic clinics [1,2,3]. Accurate radiographic diagnosis is essential, as missed or delayed fracture detection may result in malunion, nonunion, prolonged immobilization, chronic pain, and long-term functional impairment. The diagnostic challenge is particularly pronounced in the hand due to its complex anatomy and the often subtle radiographic appearance of certain fracture types, such as scaphoid fractures [4,5,6,7,8,9].

In recent years, large language models (LLMs) equipped with vision capabilities have emerged as a new class of artificial intelligence (AI) systems capable of interpreting medical images while generating natural language explanations (i.e., through natural language processing, NLP). Such systems are commonly referred to as multimodal large language models (MLLMs) when they integrate textual information with one or more additional input modalities, including visual data. Unlike conventional convolutional neural networks (CNNs), which are typically trained for narrowly defined classification or detection tasks, these MLLMs operate as general-purpose reasoning systems, raising interest in their potential role as decision-support tools in radiology. Several recent studies have explored MLLM performance in fracture detection and musculoskeletal imaging tasks, reporting heterogeneous diagnostic accuracy [10,11,12,13,14,15,16,17,18].

However, the current literature is characterized by important methodological limitations. Most studies rely on single-run evaluations, implicitly assuming deterministic behavior of MLLMs. This approach neglects the probabilistic nature of generative models, in which identical inputs can yield different outputs across repeated inferences. As a result, intra-model stability and reproducibility, which are critical prerequisites for clinical reliability, remain largely unexamined. Moreover, diagnostic accuracy is frequently reported without explicit consideration of whether correct outputs are produced consistently or merely by chance [19,20,21,22,23,24,25,26].

Another unresolved issue is the relationship between accuracy and consistency. A model may demonstrate high agreement across repeated runs while remaining systematically incorrect, or conversely produce variable but occasionally correct outputs. From a clinical perspective, these behavioral patterns carry fundamentally different risks, yet they are rarely analyzed jointly [27,28].

The present study addresses these gaps by conducting a multi-model, multi-run evaluation of MLLM performance in hand fracture detection on plain radiographs. Rather than aiming to establish population-level diagnostic validity, this work focuses on model behavior under repeated inference conditions, emphasizing the dissociation between correctness and consistency. By combining run-wise accuracy analysis, intra-model reliability metrics, case-level agreement profiles, and descriptive subgroup analyses, this study provides a structured framework for assessing the clinical trustworthiness of MLLM-based diagnostic systems.

In addition, exploratory analyses were conducted to assess whether MLLMs implicitly infer demographic attributes such as patient age and sex from radiographic images. These analyses were performed to contextualize model behavior beyond diagnostic accuracy and are reported as secondary findings.

## 2. Materials and Methods

### 2.1. Study Design and Objectives

This observational study evaluated the diagnostic behavior of four MLLMs in hand fracture detection using a repeated-run framework. The overall study design and reporting structure adhere to the Checklist for Artificial Intelligence in Medical Imaging (CLAIM) guidelines [29], within the scope applicable to the evaluation of pre-trained foundation models. The study was designed to characterize accuracy, intra-model reliability, and response stability rather than to establish clinical validation or population-level diagnostic performance.

The study objectives were:

1. To quantify diagnostic accuracy across multiple independent inference runs
2. To assess intra-model reliability using Fleiss’ kappa (κ)
3. To characterize the relationship between accuracy and consistency across models

### 2.2. Dataset and Case Selection

A total of 65 fracture-positive hand radiograph cases were selected from publicly available, curated educational radiology resources (https://radiopaedia.org, accessed on 25 December 2025) [30]. The cases were accessed and analyzed in compliance with the platform’s Terms of Use and are licensed under the Creative Commons Attribution-NonCommercial-ShareAlike 3.0 Unported License (CC BY-NC-SA 3.0). Case selection was performed manually by the authors and was guided by predefined qualitative criteria. No images were reproduced or redistributed as part of this study.

Inclusion criteria were:

(1) availability of standardized radiographic projections, specifically comprising standard two-view protocols (anteroposterior/posteroanterior and oblique/lateral) for phalangeal and metacarpal fractures, and a dedicated scaphoid series (comprising at least three projections, preferably a four-view protocol) for scaphoid fractures [31];
(2) absence of superimposed annotations (e.g., arrows, circles, or text overlays) that could serve as visual cues for the model, with the exception of standard anatomical side markers (L/R);
(3) diagnostic-quality images in web-optimized formats (JPEG/PNG), with sufficient native resolution (e.g., on the order of ~600 × 600 pixels) to allow visual assessment of cortical margins, trabecular bone structure, and fracture lines;
(4) availability of basic demographic metadata, including patient age and sex, as provided in the case description; and
(5) representation of common, clinically typical fracture patterns without rare variants, syndromic associations, or atypical imaging presentations.

The final case set comprised 30 finger (phalangeal) fractures, 30 metacarpal fractures, and 5 scaphoid fractures, reflecting a representative distribution of frequently encountered hand fracture entities in routine clinical practice. Cases were limited to fractures that were clearly identifiable on plain radiographs and did not require secondary imaging for definitive diagnosis (e.g., CT or MRI).

The study design intentionally focused exclusively on fracture-positive cases to enable detailed analysis of model behavior under conditions of confirmed pathology. This approach prioritizes the assessment of diagnostic consistency and accuracy dissociation over population-level screening performance. Specificity and negative predictive value were not assessed due to the absence of non-fracture controls. The final dataset comprised 65 fracture-positive cases covering a broad age spectrum (range: 11–80 years) with a male predominance (69.2% male, 30.8% female).

### 2.3. Models Under Evaluation

Four frontier MLLMs were evaluated, selected to represent the spectrum from proprietary US-based systems to the European open-weight paradigm:

- GPT-5 Pro (OpenAI, San Francisco, CA, USA; proprietary)
- Gemini 2.5 Pro (Google DeepMind, Mountain View, CA, USA; proprietary)
- Claude Sonnet 4.5 (Anthropic, San Francisco, CA, USA; proprietary)
- Mistral Medium 3.1 (Mistral AI, Paris, France; representing the open-source heritage)

All models were accessed via their official web interfaces using default inference settings. No API-level parameter modifications (e.g., temperature, top-p, or decoding strategies) were applied. Model versions reflect the state available in November 2024.

### 2.4. Prompting Strategy and Inference Protocol

Each model received five independent inference runs per case using an identical, standardized prompt (see Supplementary Material). The prompt instructed the model to analyze the uploaded radiograph and provide:

- A specific diagnostic classification (stating either “No fracture” or “Yes” followed by the specific fracture type, e.g., “metacarpal fracture”)
- An estimated patient age
- An inferred patient sex

To ensure independence across runs:

- Each run was conducted in a fresh session without conversational context
- No conversational memory, feedback, or adaptive prompting was permitted

Runs were treated as independent realizations of the model’s diagnostic response to identical input conditions. Evaluation was strictly stringent: Model outputs were scored as correct only if the predicted fracture type was concordant with the reference diagnosis. Consequently, misclassifications of the fracture type (e.g., correctly detecting the presence of a fracture but misidentifying a scaphoid fracture as a metacarpal fracture) were strictly scored as incorrect to rigorously penalize diagnostic imprecision and random guessing.

### 2.5. Outcome Definitions

Primary Outcomes

1. Diagnostic accuracy, defined as the mean proportion of correct classifications across five runs
2. Intra-model reliability, quantified using Fleiss’ kappa (κ) to assess agreement across repeated runs

Secondary Outcomes

1. Case-level agreement profiles, describing how often a case was correctly classified across five runs (0/5 to 5/5 correct)
2. Descriptive subgroup performance, stratified by fracture localization

### 2.6. Statistical Analysis

All statistical analyses were performed using Python 3.12 with standard statistical and data visualization libraries.

Diagnostic accuracy was summarized descriptively as the mean across five runs to characterize overall model performance, with the corresponding range (minimum–maximum) reported to illustrate intra-model variability. Intra-model reliability was assessed using Fleiss’ kappa, reflecting consistency across repeated model outputs rather than inter-observer agreement between independent raters. κ values were interpreted according to Landis and Koch [32,33,34]: <0.20 slight, 0.21–0.40 fair, 0.41–0.60 moderate, 0.61–0.80 substantial, and 0.81–1.00 almost perfect agreement.

Given the repeated-measures design and the absence of true negative cases, classical inferential testing and specificity-based metrics were not performed. Subgroup analyses were reported descriptively due to limited sample sizes, particularly for scaphoid fractures (n = 5).

### 2.7. Visualization Strategy

Results were visualized using four complementary approaches:

1. Run-wise accuracy dot plots to assess diagnostic stability
2. Accuracy–consistency dissociation matrix to relate correctness to reliability
3. Heatmaps summarizing descriptive performance by fracture subtype
4. Stacked bar charts illustrating case-level agreement profiles

This strategy was chosen to emphasize behavioral patterns and reliability characteristics rather than single-point performance estimates.

### 2.8. Exploratory Demographic Analyses

As an exploratory analysis, model outputs were additionally evaluated for implicit prediction of patient age and sex based solely on radiographic images. Age estimation accuracy was quantified using the Mean Absolute Error (MAE) between predicted and ground truth age values, averaged across five runs per model. Sex prediction accuracy was assessed as the percentage of correct classifications compared to recorded patient sex. Given the limited sample size and the lack of direct clinical relevance for fracture detection, these analyses were performed descriptively without formal hypothesis testing.

## 3. Results

### 3.1. Overall Diagnostic Accuracy and Stability

**Figure 1:**
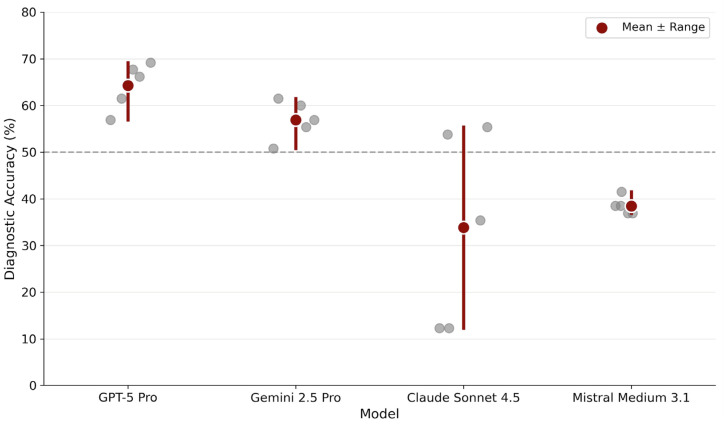
Diagnostic stability across repeated runs. Each gray dot represents accuracy from one of five independent inference runs per model. Red circles indicate mean accuracy across runs. The horizontal dashed line marks 50% accuracy as a visual reference point.

Overall diagnostic accuracy differed substantially between models (Table 1, Figure 1). GPT-5 Pro achieved the highest mean accuracy (64.3%), followed by Gemini 2.5 Pro (56.9%). Mistral Medium 3.1 (38.5%) and Claude Sonnet 4.5 (33.8%) demonstrated markedly lower performance.

Run-wise analysis revealed distinct stability profiles. GPT-5 Pro showed consistently high performance with minimal variability across runs (range: 56.9–69.2%). Gemini 2.5 Pro displayed a similarly stable profile, maintaining performance within a moderate band (range: 50.8–61.5%). In contrast, Claude Sonnet 4.5 exhibited pronounced run-to-run fluctuations (range: 12.3–55.4%), indicating probabilistic instability. Mistral Medium 3.1 displayed remarkably narrow variability (range: 36.9–41.5%) but at a substantially lower accuracy level.

### 3.2. Accuracy–Consistency Dissociation

**Figure 2:**
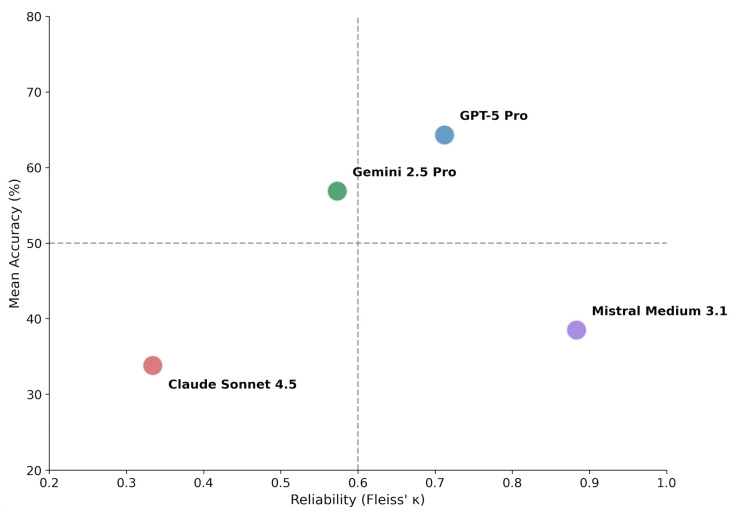
Accuracy–consistency dissociation matrix. Models are plotted according to mean diagnostic accuracy (*y*-axis) and inter-run reliability measured by Fleiss’ κ (*x*-axis). Dashed lines indicate proposed thresholds: 50% accuracy (horizontal) and κ = 0.60 corresponding to substantial agreement (vertical). The resulting quadrants characterize distinct behavioral profiles: upper-right (high accuracy, high consistency) represents ideal performance; upper-left (high accuracy, low consistency) suggests potential for improvement through consensus methods; lower-right (low accuracy, high consistency) indicates systematic error; lower-left (low accuracy, low consistency) reflects unstable diagnostic behavior.

Fleiss’ kappa analysis revealed marked differences in inter-run consistency among models (Table 2, Figure 2). The relationship between diagnostic accuracy and intra-model reliability demonstrated a critical dissociation pattern.

GPT-5 Pro combined high accuracy with substantial agreement (κ = 0.712), consistent with robust diagnostic behavior. Gemini 2.5 Pro demonstrated intermediate performance across both dimensions (κ = 0.573, moderate agreement).

Notably, Mistral Medium 3.1 exhibited very high consistency (κ = 0.883, almost perfect agreement) despite low accuracy, indicating a pattern of consistently applied but incorrect diagnostic decisions (systematic error). Claude Sonnet 4.5 showed both low accuracy and low consistency (κ = 0.334, fair agreement), reflecting unstable and non-deterministic diagnostic behavior.

Using thresholds of 50% accuracy and κ = 0.60 to delineate quadrants (Figure 2), the four models distributed across distinct behavioral profiles: GPT-5 Pro occupied the upper-right quadrant (high accuracy, high consistency). Gemini 2.5 Pro positioned in the upper-left region with above-average accuracy but only moderate consistency. Mistral Medium 3.1 fell in the lower-right quadrant (low accuracy, high consistency), representing systematic error. Claude Sonnet 4.5 occupied the lower-left quadrant (low accuracy, low consistency), reflecting unstable diagnostic behavior.

### 3.3. Descriptive Subgroup Performance

**Figure 3:**
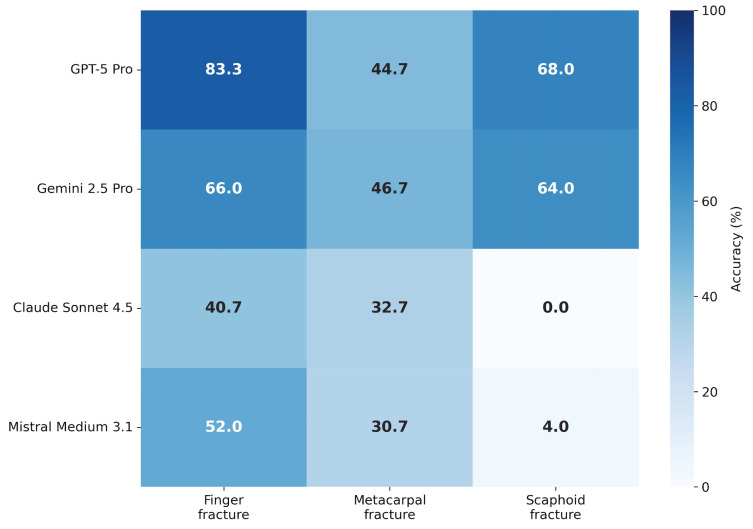
Descriptive performance by fracture subtype. Heatmap displaying mean diagnostic accuracy stratified by fracture type (finger, metacarpal, scaphoid). Color intensity corresponds to accuracy percentage (darker = higher accuracy). Values are averaged across all five inference runs per model.

Model performance varied by fracture type (Table 3, Figure 3). Finger fractures were detected with the highest accuracy across most models, with GPT-5 Pro achieving 83.3% and Gemini 2.5 Pro reaching 66.0%. Metacarpal fractures proved more challenging, with accuracies ranging from 30.7% (Mistral Medium 3.1) to 46.7% (Gemini 2.5 Pro).

Scaphoid fractures (n = 5) represented the most difficult subgroup. While GPT-5 Pro and Gemini 2.5 Pro achieved moderate performance (68.0% and 64.0%, respectively), Claude Sonnet 4.5 failed entirely (0.0% accuracy across all runs), and Mistral Medium 3.1 achieved only 4.0%.

### 3.4. Case-Level Agreement Profiles

Claude Sonnet 4.5 showed predominantly unstable case-level behavior across runs, whereas Mistral Medium 3.1 demonstrated a polarized pattern characterized by consistently correct or consistently incorrect classifications.

### 3.5. Exploratory Demographic Attribute Inference

As a secondary analysis, model outputs were examined for implicit inference of patient age and sex across all five runs (n = 325 observations per model). Performance was generally poor.

For age estimation, GPT-5 Pro achieved the lowest Mean Absolute Error (MAE) of 12.98 ± 9.81 years, whereas other models exhibited larger deviations: Gemini 2.5 Pro (16.00 ± 12.02 years), Mistral Medium 3.1 (17.72 ± 14.07 years), and Claude Sonnet 4.5 (18.28 ± 12.98 years). Mean age estimation errors exceeded one decade across all evaluated models.

Sex prediction accuracy was remarkably low, ranging from 48.9% (Gemini 2.5 Pro) to 62.8% (GPT-5 Pro). Notably, given the male predominance in the study cohort (69.2%), a naive classifier predicting “male” for every case would have achieved higher accuracy than any of the evaluated models. Claude Sonnet 4.5 (49.2%) and Mistral Medium 3.1 (49.2%) approximated chance-level performance.

## 4. Discussion

### 4.1. Accuracy–Consistency Dissociation as a Core Behavioral Property

The central finding of this study is the marked dissociation between diagnostic accuracy and intra-model consistency, as illustrated in the Accuracy–Consistency Dissociation Matrix (Figure 2). This observation challenges a common implicit assumption in current AI evaluation paradigms, namely that higher diagnostic accuracy or reproducibility necessarily implies more reliable or clinically trustworthy behavior [35].

GPT-5 Pro occupied the quadrant of both high accuracy and substantial inter-run agreement (κ = 0.71), representing the most favorable behavioral profile among the evaluated systems. Gemini 2.5 Pro demonstrated intermediate performance across both dimensions (κ = 0.57), suggesting partially stable but not fully robust diagnostic behavior. In contrast, Mistral Medium 3.1 exhibited a clinically concerning pattern: despite achieving almost perfect intra-model agreement (κ = 0.88), its diagnostic accuracy remained low (~38%). This constellation reflects a form of systematic error characterized by highly reproducible incorrect predictions, a pattern commonly referred to in the MLLM/LLM literature as confident hallucination. In a clinical context, such high reproducibility may be misinterpreted as confidence or validity, thereby posing substantial risk [11,36].

Claude Sonnet 4.5 demonstrated the opposite failure mode, with low overall accuracy combined with pronounced run-to-run variability (accuracy range: 12.3–55.4%; κ = 0.33; Figure 1). This probabilistic instability undermines the reliability of individual outputs and precludes dependable clinical use, even when occasional correct predictions are observed.

### 4.2. Diagnostic Stability Beyond Single-Run Accuracy

**Figure 4:**
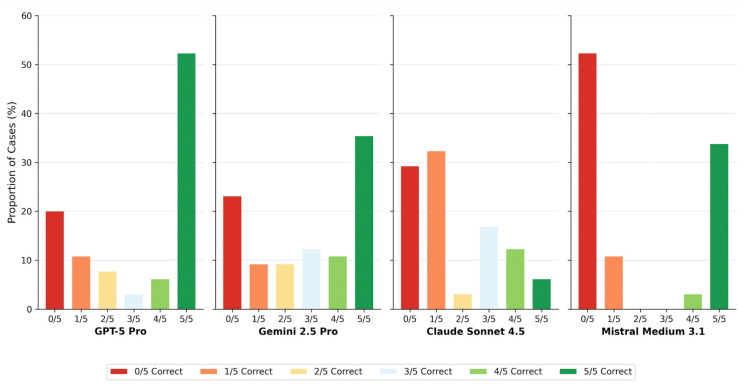
Case-level agreement profiles. Bar chart showing the distribution of consensus categories for each model. Colors range from red (0/5 runs correct) through yellow (2–3/5) to green (5/5 runs correct). The proportion of cases achieving full consensus (5/5 or 0/5) versus mixed results (1–4/5) characterizes model decision stability.

Run-wise accuracy analysis (Figure 1) and case-level agreement profiles (Figure 4) underscore that single-point accuracy metrics provide an incomplete characterization of MLLM diagnostic performance. Traditional AI benchmarks often rely on aggregate metrics derived from a single inference pass, implicitly assuming deterministic model behavior. This assumption does not hold for generative models, where repeated inferences from identical inputs may yield divergent outputs [19,20,21,22,23,24,25,26].

Case-level agreement profiles further elucidate these behavioral differences. GPT-5 Pro achieved full consensus (5/5 correct runs) in 46.2% of cases, whereas Claude Sonnet 4.5 reached this level in only 15.4%. Conversely, complete diagnostic failure (0/5 correct runs) occurred in over half of all cases for Claude Sonnet 4.5 (53.8%), compared with 21.5% for GPT-5 Pro (Table 4, Figure 4). The distribution of intermediate agreement levels differed markedly across models, revealing fundamentally different decision stability profiles that are obscured when performance is summarized solely by mean accuracy.

### 4.3. Anatomical Complexity and Task-Specific Limitations

Descriptive subgroup analysis demonstrated pronounced performance differences across fracture localizations (Table 3, Figure 3). While phalangeal fractures were detected relatively reliably by the top-performing model (>80% accuracy for GPT-5 Pro), scaphoid fractures represented a major challenge. Despite the small subgroup size (n = 5), which necessitates cautious interpretation, the near-complete failure of Claude Sonnet 4.5 (0.0% accuracy) and Mistral Medium 3.1 (4.0% accuracy) suggests a fundamental limitation in integrating complex, overlapping carpal anatomy across multiple two-dimensional radiographic projections.

These findings are consistent with known challenges in automated scaphoid fracture detection and highlight the current performance gap between general-purpose MLLMs and specialized CNNs optimized for musculoskeletal imaging. While MLLMs offer flexibility and broad reasoning capabilities, they currently lack the task-specific inductive biases required for reliable detection of subtle orthopedic pathologies [37].

### 4.4. Demographic Attribute Inference and Diagnostic Performance

Exploratory analyses revealed that all evaluated models performed poorly in inferring patient age and sex from hand radiographs (Table 5). MAE in age estimation exceeded one decade across all systems, and sex prediction accuracy approximated chance level for most models. Importantly, demographic inference performance did not correlate with diagnostic accuracy in fracture detection tasks.

These findings serve as a negative control rather than a performance benchmark. The inability of MLLMs to reliably infer demographic attributes from hand radiographs suggests that fracture detection is not driven by incidental correlations with age- or sex-related visual cues. Accordingly, correct fracture detection in this study does not appear to depend on implicit demographic inference, and demographic attribute prediction should not be interpreted as a proxy for diagnostic competence or clinical reasoning capability.

### 4.5. Limitations and Future Directions

This study has several methodological limitations that can be grouped into four main domains: data heterogeneity and cohort composition, scalability and evaluation constraints, benchmarking scope, and model-related sources of variability. These limitations primarily reflect current practical and conceptual constraints in evaluating MLLMs under real-world conditions.

1. The radiographic cases were derived from heterogeneous sources [30] and were not acquired under standardized imaging protocols. Differences in imaging devices, acquisition parameters, projection angles, and post-processing settings reflect real-world variability but limit strict control over image quality and comparability. Consequently, model performance may be influenced by uncontrolled technical factors inherent to the source material.
2. The study design exclusively included fracture-positive cases. While this approach was intentionally chosen to enable focused analysis of diagnostic behavior, consistency, and error patterns under conditions of confirmed pathology, it necessarily limits the scope of inference. In particular, specificity, false-positive rates, and population-level screening performance could not be assessed. Future investigations should therefore incorporate substantially larger and balanced datasets that include both fracture-positive and fracture-negative cases.

However, performing such large-scale, balanced evaluations with repeated inference runs is not feasible through standard web-based user interfaces. Repeated manual uploading of thousands of radiographic images across multiple runs would be impractical. Consequently, reproducible large-scale repeated-run analyses require programmatic access to MLLMs via application programming interfaces (APIs). Such API-based evaluation deviates from typical end-user interaction paradigms and introduces additional constraints related to token-based billing, scalability, and comparability with real-world clinical usage. Accordingly, the present study intentionally reflects real-world web-interface usage rather than optimized API-based deployments, prioritizing ecological validity over scalability.

3. The sample size, particularly for anatomically complex subgroups such as scaphoid fractures, was limited. As a result, subgroup analyses were descriptive in nature and should not be interpreted as definitive performance estimates. Larger, pathology-stratified cohorts will be required to robustly assess model behavior across less frequent but clinically critical fracture types.
4. This study evaluated MLLMs in isolation and did not include direct comparisons with human experts or with specialized CNN–based fracture detection systems. Benchmarking against experienced radiologists and task-specific CNN tools represents an important next step to contextualize the observed MLLM behavior within established clinical workflows. Comparative evaluations involving human experts, CNN-based systems, and MLLMs would allow assessment of complementary strengths, failure modes, and potential hybrid decision-support strategies.
5. The present analysis focused on diagnostic accuracy and intra-model consistency across repeated inference runs. While these metrics capture important aspects of reliability, they do not fully reflect clinical utility. Future benchmarking frameworks should incorporate additional dimensions, including time-to-decision, workflow integration, cognitive load reduction, and robustness under realistic deployment constraints. In particular, comparisons between human experts, CNN-based systems, and MLLMs should consider diagnostic performance in relation to time expenditure and operational efficiency rather than accuracy alone.
6. Generative models introduce unique sources of variability related to stochastic decoding processes. Parameters such as temperature, sampling strategies, and system-level nondeterminism may substantially influence output stability. In many real-world deployments, especially web-based interfaces, such parameters are not fully transparent or controllable by the user. This limits reproducibility and complicates fair benchmarking across systems. Future studies should therefore prioritize controlled evaluation environments that allow systematic regulation of stochastic inference settings.
7. Binary fracture detection alone captures only a limited aspect of clinical decision-making. From a clinical perspective, additional descriptive elements, including fracture classification, displacement, extent, and contextual interpretation, would likely be required before such systems could be considered for meaningful decision support.
8. Model performance represents a temporal snapshot tied to specific model versions and deployment configurations. Given the rapid evolution of multimodal architectures, frequent updates, and opaque system changes, longitudinal assessments will be necessary to determine whether observed behavioral patterns remain stable over time or shift with subsequent model iterations.
9. In addition, evolving model-level safety and moderation policies introduce an external source of variability that may affect the reproducibility of diagnostic evaluations across model versions and time.

### 4.6. Practical Considerations: Terms of Use, Cost Structure, and Workflow Constraints

All evaluated MLLMs are offered under provider-specific terms of use and are available in both free and multiple paid access tiers. Basic personal usage is typically covered by low-cost subscription models with strict limitations on image uploads and token consumption, whereas professional or high-volume usage is associated with substantially higher costs and, in some cases, adaptive pricing models. Precise cost comparisons were not performed, as pricing structures are dynamic, subject to frequent changes, and strongly dependent on the user’s region.

From a workflow perspective, access via web-based interfaces requires manual image upload and prompt submission for each inference, which is not suitable for frequent or large-scale use. Such interfaces are not designed for batch processing or systematic image evaluation, as required in research settings or clinical workflows. Programmatic access via APIs would therefore be a prerequisite for scalable deployment. In addition, current MLLMs increasingly restrict responses to medical and diagnostic queries, frequently issuing safety-related refusals or deferring to human clinicians, which represents a further practical limitation for reproducible medical image evaluation.

## 5. Conclusions

In this multi-run evaluation of MLLMs for hand fracture detection, diagnostic accuracy and intra-model consistency emerged as distinct and partially dissociated performance dimensions. GPT-5 Pro demonstrated the most favorable balance of accuracy (64.3%) and stability (κ = 0.71) among the evaluated systems, whereas other models exhibited critical failure modes ranging from systematic error with high reproducibility (Mistral Medium 3.1) to pronounced probabilistic instability (Claude Sonnet 4.5).

Key Diagnostic Insights

- Dissociation of Accuracy and Consistency: High intra-model agreement must not be equated with diagnostic correctness, as reproducibility may conceal consistently erroneous reasoning or unstable probabilistic behavior.
- Task-Dependent Performance: Model performance varied substantially by anatomical complexity, with relatively robust detection of phalangeal fractures but persistent limitations in complex carpal anatomy such as scaphoid fractures.
- Demographic Independence: Diagnostic performance was independent of implicit age or sex inference, indicating that fracture detection was not driven by incidental demographic visual cues.

Research Roadmap for Future Evaluation

- Reliability-Aware Evaluation: Future studies should routinely incorporate repeated-run analyses and reliability metrics alongside single-run accuracy to distinguish robust reasoning from stable or unstable error patterns.
- Dataset Expansion: Larger and balanced datasets including fracture-negative cases are required to assess specificity and population-level screening performance.
- Clinical Benchmarking: Systematic comparisons with task-specific CNN-based systems and human experts, ideally using controlled API-based evaluation environments, are necessary to contextualize MLLM behavior within real-world clinical workflows.
- Longitudinal Stability Monitoring: Given the rapid evolution of multimodal architectures, longitudinal assessments are necessary to determine if diagnostic behavior remains stable over subsequent model iterations and changing safety policies.

## Figures and Tables

**Table 1:** Diagnostic accuracy across five inference runs per model (n = 65 cases).

Model	Run 1	Run 2	Run 3	Run 4	Run 5	Mean (Range)
GPT-5 Pro	61.5	69.2	66.2	67.7	56.9	64.3 (56.9–69.2)
Gemini 2.5 Pro	61.5	50.8	56.9	60.0	55.4	56.9 (50.8–61.5)
Claude Sonnet 4.5	12.3	55.4	35.4	12.3	53.8	33.8 (12.3–55.4)
Mistral Medium 3.1	38.5	41.5	36.9	36.9	38.5	38.5 (36.9–41.5)

Values represent percentage accuracy. Range indicates minimum–maximum across five runs.

**Table 2:** Inter-run reliability (Fleiss’ κ) and summary statistics.

Model	Fleiss’ κ	Interpretation	Accuracy Range (%)
GPT-5 Pro	0.712	Substantial	56.9–69.2
Gemini 2.5 Pro	0.573	Moderate	50.8–61.5
Claude Sonnet 4.5	0.334	Fair	12.3–55.4
Mistral Medium 3.1	0.883	Almost perfect	36.9–41.5

Interpretation according to Landis and Koch (1977): <0.20 slight, 0.21–0.40 fair, 0.41–0.60 moderate, 0.61–0.80 substantial, 0.81–1.00 almost perfect [32,33,34].

**Table 3:** Diagnostic accuracy by fracture subtype (descriptive analysis).

Model	Finger (n = 30)	Metacarpal (n = 30)	Scaphoid (n = 5)
GPT-5 Pro	83.3	44.7	68.0
Gemini 2.5 Pro	66.0	46.7	64.0
Claude Sonnet 4.5	40.7	32.7	0.0
Mistral Medium 3.1	52.0	30.7	4.0

Values represent mean accuracy (%) across 5 runs.

**Table 4:** Case-level agreement distribution of correct agreement across five runs.

Model	0/5	1/5	2/5	3/5	4/5	5/5
GPT-5 Pro	13	7	5	2	4	34
Gemini 2.5 Pro	15	6	6	8	7	23
Claude Sonnet 4.5	19	21	2	11	8	4
Mistral Medium 3.1	34	7	0	0	2	22

Values represent the number of cases (n = 65) classified correctly in 0–5 out of five independent runs.

**Table 5:** Exploratory results for demographic attribute inference.

Model	Age MAE (Years)	Sex Accuracy (%)
GPT-5 Pro	12.98 ± 9.81	62.8
Gemini 2.5 Pro	16.00 ± 12.02	48.9
Claude Sonnet 4.5	18.28 ± 12.98	49.2
Mistral Medium 3.1	17.72 ± 14.07	49.2

MAE = Mean Absolute Error (mean ± SD across n = 325 observations per model). Majority-class baseline for sex prediction: 69.2% (male).

## Data Availability

The radiographic images used in this study were obtained from publicly available educational resources and were used solely as input stimuli for model evaluation. In accordance with applicable content licenses and third-party copyright, the original image data are not redistributed. Derived data, including aggregated performance metrics and model outputs, are available from the corresponding author upon request.

## Supplementary Materials

The following supporting information can be downloaded at: https://www.mdpi.com/article/10.3390/diagnostics16030424/s1, Supplementary Material: Full standardized zero-shot prompt used for all inference runs.

## Abbreviations

The following abbreviations are used in this manuscript:

AI	Artificial Intelligence
API	Application Programming Interface
CNN	Convolutional Neural Network
κ	Fleiss’s Kappa (inter-rater reliability coefficient)
LLM	Large Language Model
MAE	Mean Absolute Error
MLLM	Multimodal Large Language Model
NLP	Natural Language Processing
SD	Standard Deviation
