# Supplementary material for: Diagnostic Accuracy and Stability of Multimodal Large Language Models for Hand Fracture Detection: A Multi-Run Evaluation on Plain Radiographs
Source: Diagnostics (Basel). 2026 Feb 1;16(3):424. doi: 10.3390/diagnostics16030424 (PMC12897326; doi:10.3390/diagnostics16030424)
Supplement: Supplementary file 1 [file diagnostics-16-00424-s001.zip › diagnostics-4109629-supplementary.pdf]

Prompt Used for All Inference Runs

Context You are provided with a plain radiograph of the human hand obtained in a clinical setting. The image shows standard diagnostic projections used for fracture assessment.

Objective Assess the radiograph for the presence of a fracture and report the findings in a structured manner.

Style Respond in a concise, factual, and clinically neutral tone. Do not provide differential diagnoses, recommendations, or disclaimers.

Task Based solely on the visual information in the uploaded radiograph, perform the following tasks:

- Determine whether a fracture is present.
  - If no fracture is visible, respond: "No fracture detected."
  - If a fracture is present, respond: "Yes – [specify fracture type]," using one of the following categories: phalangeal fracture, metacarpal fracture, or scaphoid fracture.
- Estimate the patient's age in years based on the radiographic appearance.
- Infer the patient's sex (male or female) based on the radiographic appearance.

Audience No explanation or justification is required.

Response Format Provide your response strictly in the following format, without additional commentary:

Fracture: [No fracture detected / Yes – fracture type]

Estimated age: [number]

Inferred sex: [male / female]
